# Are human endogenous retroviruses triggers of autoimmune diseases? Unveiling associations of three diseases and viral loci

**DOI:** 10.1007/s12026-015-8671-z

**Published:** 2015-06-20

**Authors:** Bjørn A. Nexø, Palle Villesen, Kari K. Nissen, Hanne M. Lindegaard, Peter Rossing, Thor Petersen, Lise Tarnow, Bettina Hansen, Tove Lorenzen, Kim Hørslev-Petersen, Sara B. Jensen, Shervin Bahrami, Maria Lajer, Kathrine L. M. Schmidt, Hans-Henrik Parving, Peter Junker, Magdalena J. Laska

**Affiliations:** Department of Biomedicine, Aarhus University, Bartholins Alle’ 6, 8000 Århus C, Denmark; Bioinformatics Research Center, Aarhus University, 8000 Århus C, Denmark; Department of Rheumatology, Odense University Hospital, 5000 Odense C, Denmark; Steno Diabetes Center, 2820 Gentofte, Denmark; NNF CBMR, University of Copenhagen, 1017 Copenhagen, Denmark; Faculty of Health, Aarhus University, 8000 Århus C, Denmark; Department of Neurology, Aarhus University Hospital, 8000 Århus C, Denmark; Nordsjaellands Hospital, 3400 Hillerod, Denmark; Department of Rheumatology, Vejle Hospital, 71000 Vejle, Denmark; King Christian 10th Hospital for Rheumatic Diseases, 6300 Graasten, Denmark; Department of Clinical Medicine, Aarhus University, 8000 Århus C, Denmark; Department of Medical Endocrinology, Rigshospitalet, University of Copenhagen, 1017 Copenhagen, Denmark

**Keywords:** Multiple sclerosis, Type 1 diabetes mellitus, Rheumatoid arthritis, Synergy, Single nucleotide polymorphisms

## Abstract

Autoimmune diseases encompass a plethora of conditions in which the immune system attacks its own tissue, identifying them as foreign. Multiple factors are thought to contribute to the development of immune response to self, including differences in genotypes, hormonal milieu, and environmental factors. Viruses including human endogenous retroviruses have long been linked to the occurrence of autoimmunity, but never proven to be causative factors. Endogenous viruses are retroviral sequences embedded in the host germline DNA and transmitted vertically through successive generations in a Mendelian manner. In this study by means of genetic epidemiology, we have searched for the involvement of endogenous retroviruses in three selected autoimmune diseases: multiple sclerosis, type 1 diabetes mellitus, and rheumatoid arthritis. We found that at least one human endogenous retroviral locus was associated with each of the three diseases. Although there was a significant overlap, most loci only occurred in one of the studied disease. Remarkably, within each disease, there was a statistical interaction (synergy) between two loci. Additional synergy between retroviral loci and human lymphocyte antigens is reported for multiple sclerosis. We speculate the possibility that recombinants or mixed viral particles are formed and that the resulting viruses stimulate the innate immune system, thereby initiating the autoimmune response.

**Electronic supplementary material:**

The online version of this article (doi:10.1007/s12026-015-8671-z) contains supplementary material, which is available to authorized users.

## Introduction

The autoimmune diseases (AID) are a diverse group of conditions characterized by abnormal immune reactivity in association with autoreactive B and T cell responses. More than 80 AID have been identified. The most common of these diseases include systemic lupus erythematosus, multiple sclerosis (MS), type 1 diabetes mellitus (T1DM), autoimmune thyroid disease, myasthenia gravis, scleroderma, and rheumatoid arthritis (RA) [1]. MS, RA, and T1DM are all characterized by the body’s immune response being directed against its own tissues, causing prolonged inflammation and subsequent tissue destruction. Cell populations of particular importance in these three conditions include oligodendrocytes and possibly neuronal cells, pancreatic islet β-cells, and synovial cells, respectively. Multiple factors are thought to contribute to the development of immune responses to self, including differences in genotypes, hormonal milieu, and environmental triggers [2, 3]. The significance of environmental effectors is underscored by the generally low concordance rates of major AID in monozygotic twins [3].

There is abundant animal experimental evidence that viruses can play a causative role in chronic AID [4, 5]. Possible mechanisms including alterations in target cells, alterations in immune cells, immune responses against the determinant shared by host and virus, and cross-reaction between idiotype and the antiviral antibody and respective autoantigens have been suggested. Much of what we know about these mechanisms has come primarily from studies in animal models, but there is compelling epidemiologic evidence that virus infection in humans predisposes one to AID [4, 6]. However, no known virus has been proven to regularly induce or promote autoimmune disorders.

Endogenous retroviruses are found in all vertebrates including humans. Their origins are thought to be prehistoric infection by exogenous retroviruses of germline cells resulting in the integration of the retroviral genome (the provirus) into the genome of the cells. Thus, endogenous retroviruses are integral parts of animal genomes and are transmitted vertically through successive generations in a Mendelian manner.

Endogenous retroviruses are peculiar in that they span the immunological divide between self and foreign. On the one hand, they are part of the organism itself, encoded in the chromosomes. On the other hand, they share properties with infectious agents and any produced viral particles by such endogenous viruses are not much different from particles of exogenous infectious agents. Therefore, it can be expected that virions derived from endogenous retroviruses will be able to trigger several pattern recognizers of the innate immune system resulting in induction of autoimmunity.

Retroviral particles are known to be immune suppressive. This is primarily the result of immunosuppressive protein sequences found in the retroviral surface (envelope) protein, which increase the viability of the virions in the host organism. Such immunosuppressive activities are also found in human endogenous retroviruses (HERVs) [7] and might have been adapted by placental mammals as a means of protecting embryos from the maternal immune system [8]. Indeed, the endogenous retroviruses play a crucial role in formation of mammalian placenta.

Most HERVs are defective through accumulation of mutations during their millions of years as proviruses in the genome, although very few still contain open reading frames (ORFs) that are free from deletion and nonsense mutations [9]. Around 50 loci are able, with one or two mutations per viral gene, to encode one or more viral proteins. For example, HERV-Ks are the biologically most active human endogenous virus family with HERV-K sequences coding for all necessary retroviral proteins. While HERVs have generally been thought to be “fossils” of formerly infectious viruses that have since become incapable of replication, recent data have suggested that there may in fact be situations in which HERV-K may be able to replicate competently within their human host. Some of these recently integrated proviruses are responsible for synthesis of retroviral particles that can be detected in teratocarcinoma [10, 11] and melanoma-derived cell lines [12], and possibly in human placenta [13]. Despite this activity, no functional provirus able to produce infectious particles has yet been described. In mice, recombination between replication-defective ERV loci can lead to replication-component loci [14, 15]. This provides a plausible model for the reconstitution of mobile and integration-competent HERVs within the genome [16].

Genome-wide association studies (GWAS) have led to the identification of hundreds of common single nucleotide polymorphisms (SNPs) involved in susceptibility to autoimmune disorders. However, repetitive sequences which are characteristic for HERV sequences have not been adequately inspected through GWAS. In this study, we searched for evidence implicating HERVs in the etiology of complex AID with particular reference to MS, T1DM, and RA. We adopted a classical genetic epidemiology approach rather than direct virology techniques, because we wanted to circumvent the difficult issue of viruses as innocent bystanders present in diseased tissue and organs. Moreover, a genetic approach allows for direct identification of the endogenous viral locus, and not just the type of virus. We report here that distinctive markers in or near endogenous retroviral loci are correlated with the different diseases. Remarkably, in all three diseases, there was a statistically significant interaction between two viral loci, suggesting that the gene products act synergistically, e.g., through recombination or complementation.

## Materials and methods

### Human samples

The studies were approved by the Research Ethics Committees of the Capital Region, the Central Denmark region, and Region of Southern Denmark. All study individuals gave informed written and oral consent. MS was identified according to recommended diagnostic criteria for MS: guidelines from the International Panel [17]. T1DM was defined according to World Health Organization criteria. RA was diagnosed according to the American College of Rheumatology 1987 revised criteria for RA [18]. Blood was drawn and peripheral blood mononuclear cells (PBMCs) were isolated and cryopreserved for later DNA purification. A record of gender was kept for all participants. MS patients (350 persons) were recruited at the Department of Neurology, Aarhus University Hospital, Denmark, over a period of several years. T1DM patients (1183 persons) were all of Scandinavian descent, and samples were collected at Steno Diabetes Center, Copenhagen, Denmark [19]. The RA patients (710 persons) were recruited from three university hospital outpatient clinics in Southern Jutland and Funen, Denmark [20]. Gender distribution was provided for subcohorts. Control samples were collected among medical students at Aarhus University, Denmark. DNA was purified immediately according to standard laboratory protocol. In addition, 40 control samples originating from healthy Danish blood donors from Aarhus were included. For T1DM, an extra set of healthy controls were included. These are healthy blood donors from Odense, Denmark. However, information about the gender was missing in one subgroup.

### Position of endogenous viruses

The 51 loci were chosen for having at most two stop codons or frame-shift mutations in a viral genome (see Supplemental Table 1). The positions of the endogenous viral loci correspond to NCBI Annotation 106.

### Single nucleotide polymorphisms (SNPs)

SNPs were genotyped using PCR and mass-spectrometry-based Sequenom^8^ platform (San Diego, CA) using conditions as previously described [21]. Tested SNPs were localized in or near the 51 HERV loci (<10 kb). A list of the SNPs originally tested is given in the supplemental information in [21]. Positions of the SNPs were verified using the GRCh37.p13 assembly. Some markers were later rejected, either because there was too many missing values (>20 %), because the SNP was monotonic or nearly so, or because scrutiny of the Sequenom readouts revealed it to be unreliable. In the final analysis, 284, 130, and 116 SNPs were employed for MS, T1DM, and RA, respectively.

### Statistics

The genetic data and gender information were stored and processed in SPSS (IBM, Armonk, NY). Associations of single SNPs with disease were analyzed using the “crosstabs” procedure. The frequency of genotypes provided the *P* value for association (*P*_G_). To correct for multiplicity of testing, we employed Bonferroni correction, i.e., the *P* values were multiplied with the number of tests performed to give the corrected *P* value (*P*_B_). For the ANOVA, controls were coded as 0 and cases as 1 in SPSS, and the analyses were performed as a univariate linear model using type 3 sums of squares. The significance of the product term was used as measure of synergy (*P*). For all cases, analyses involving SNPs on the X chromosome were gender-separated and the resulting *P* values combined using Fisher’s method. In table presenting odds ratios, confidence intervals for the alleles, and the probability of the allele distribution (not to be confused with *P*_G_), results were calculated in Excel (Microsoft, Redmond, WA).

## Results

The human genome as it is currently known contains 51 endogenous retroviral loci with one or more intact or near-intact genes [21]. Here, we concentrated the analysis on these loci. A list of these endogenous viruses is presented as Supplemental Table 1. SNPs in or near these endogenous retroviral loci were reported previously [21].

### Multiple sclerosis (MS)

We have previously reported that MS is associated with the C-allele of the SNP rs391745 (*P*_G_ = 3.1 × 10^−5^; *P*_B_ = 0.009) located upstream of the retroviral locus HERV-Fc1 (Chr X position 97,841,482–97,849,424) [22] [OR_male_ (CI) 3.73 (2.02–6.88); OR_female_ (CI) 2.03 (1.40–2.94)] [21]. Importantly, HERV-Fc1 has a *pol* region, which is interrupted by two stop codons and a frame-shift mutation, suggesting complementation by another locus. Subsequent search of the 51 HERV loci for intact or near-intact *pol* reading frame led to the finding of 16 such viruses. Interestingly, among these, we identified a HERV-K13 (Chr 19 position 21,880,252–21,890,542), which had given the next highest association with MS [10].

**Fig. 1:**
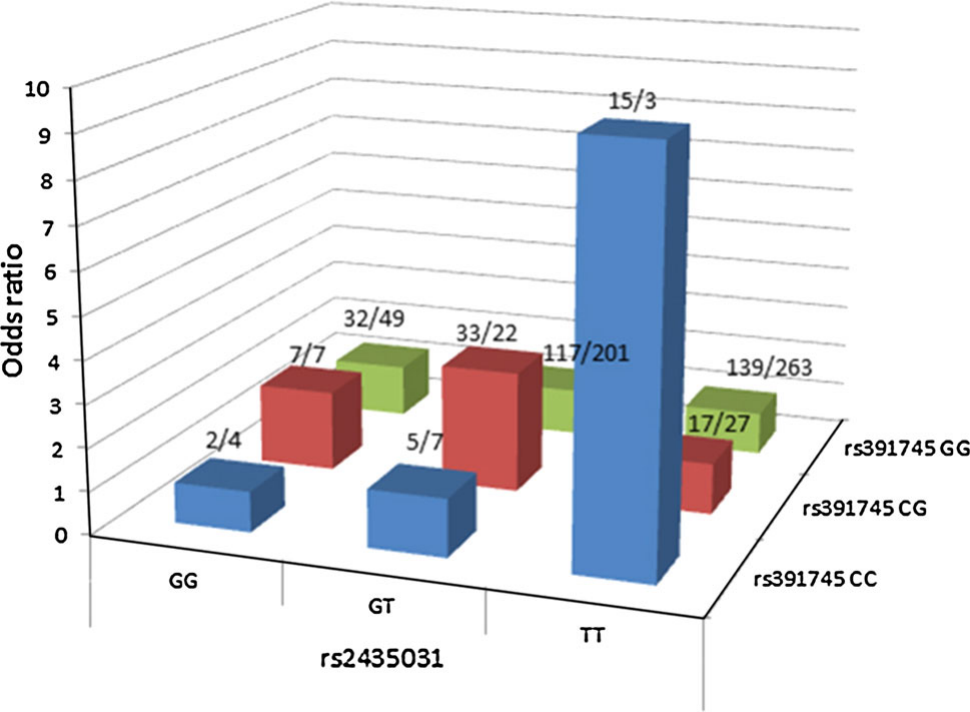
Synergy between markers of HERV-K13 and HERV-Fc1 in multiple sclerosis. For simplicity, men were depicted as homozygous although they really are hemizygous for rs391745. The *columns* represent the odds ratio for easy genotype combination, while the numbers over each column give the number of cases and controls for each genotype combination

Among six markers near HERV-K13 [23], four showed signs of interaction with rs391745/Fc1 with a rs2435031/K13 giving the highest interaction (*P* = 0.02). To illustrate the interaction, we depicted the odds ratios for all nine genotype combinations of rs2435031/K13 and rs391745/Fc1, calculated against the most common genotype, rs391745/Fc1^GG^ rs2435031/K13^TT^. For simplicity, we pooled men and women and calculated men as homozygous for rs391745/Fc1 (Fig. 1). The effect of the risk genotype rs2435031/K13^TT^ rs391745/Fc1^CC^ was strong, and the combined odds ratio for the partially and fully heterozygote carriers was also increased [OR (95 %CI) = 1.86 (1.21–2.84); *P* = 0.004].

Reciprocally, we searched 43 markers in and around HERV-Fc1 for the best interaction with rs2435031/K13. The most significant SNP was rs318138/Fc1, giving a *P*_I_ = 0.0009. It is not clear how to correct for the multiplicity of testing in this tiered approach, but this is a very strong interaction. Table 1 illustrates the complete ANOVA for this combination.

**Table 1:** Two-way ANOVA of synergy between markers of HERV-K113 and HERV-Fc1 in MS

Gender	Source	Type III sum of squares	Degrees of freedom	Mean square	*F*	*P* value
Female	Model	6.520	8	0.815	3.638	0.000
	Intercept	9.395	1	6.395	28.549	0.000
	rs2435031	0.522	2	0.261	1.164	0.313
	rs318138	1.435	2	0.717	3.203	0.041
	rs2435031 × rs318138	2.451	4	0.613	2.735	***0.028***
	Error	126.784	566	0.224		
	Total	210.000	575			
	Corrected total	133.304	574			
Male	Model	5.094	5	1.019	4.432	0.001
	Intercept	15.992	2	15.922	69.220	0.000
	rs2435031	2.459	3	1.230	5.346	0.005
	rs318138	0.493	1	0.493	2.142	0.144
	rs2435031 × rs318138	2.796	2	1.398	6.077	***0.003***
	Error	71.074	309	0.230		
	Total	129.000	315			
	Corrected total	76.171	314			
Combined	rs2435031 × rs318138					***0.0009***

*P* values for two-way interactions between rs2435031 and rs318138 are shown in bold and italics

The human lymphocyte antigens (*HLA*) have repeatedly been implicated as factors in MS [24]. The associated allele is HLA-DRB1*1501, tagged by rs3135388/HLA [25]. A gender-separated three-way ANOVA with rs2435031/K13, rs318138/Fc1, and rs3135388/HLA showed that the *P* value associated with the triple product term was significant (*P*_I_ = 0.02), indicating that the *HLA* marker interacts with both viral markers simultaneously. The *P* value of the overall model was very low (*P* = 6 × 10^−13^; Table 2).

**Table 2:** Three-way ANOVA of synergy between markers of HERV-K13, HERV-Fc1, HLA in MS

Gender	Source	Type III sum of squares	Degrees of freedom	Mean square	*F*	*P* value
Female	Corrected model	16.236	20	0.812	3.833	0.000
	Intercept	6.995	1	6.995	33.031	0.000
	rs2435031	0.735	2	0.367	1.735	0.177
	rs318138	0.102	2	0.051	0.240	0.787
	rs3135388	0.179	2	0.089	0.422	0.656
	rs2435031 × rs318138	1.334	4	0.333	1.574	0.180
	rs2435031 × rs3135388	1.954	4	0.488	2.306	0.057
	rs318138 × rs3135388	1.719	3	0.573	2.705	0.045
	rs2435031 × rs318138 × rs3135388	1.781	3	0.594	2.803	***0.039***
	Error	110.758	523	0.212		
	Total	202.000	544			
	Corrected total	126.993	543			
Male	Corrected model	13.060	14	0.933	4.488	0.000
	Intercept	13.074	1	13.074	62.898	0.000
	rs2435031	1.008	2	0.504	2.425	0.090
	rs318138	0.499	1	0.499	2.399	0.122
	rs3135388	2.549	2	1.275	6.132	0.002
	rs2435031 × rs318138	1.198	2	0.599	2.882	0.058
	rs2435031 × rs3135388	1.159	4	0.290	1.394	0.236
	rs318138 × rs3135388	0.144	2	0.072	0.346	0.708
	rs2435031 × rs318138 × rs3135388	0.610	1	0.610	2.934	***0.088***
	Error	59.655	287	0.208		
	Total	122.000	302			
	Corrected total	72.715	301			
Combined	rs2435031 × rs318138 × rs3135388					***0.02***

*P* values for three-way interactions between rs2435031, rs318138 and rs3135388 are shown in bold and italics

### Type 1 diabetes mellitus (T1DM)

We identified one SNP as showing association with T1DM, rs7650483 near HERV-K119 encoding *gag* and *env* located on chromosome 3 (position 113,024,277–113,033,435) (T allele associated with disease, OR (CI)1.25 (1.08–1.43); *P*_G_ = 0.0004). The association with T1DM was significant after Bonferroni correction (*P*_B_ = 0.05). Two other SNPs were also found to be associated with disease although they could not withstand strength of Bonferroni corrections. Subsequent analysis of these SNPs revealed synergy between rs7650483/K119 and rs11172544/K106 located on Chr. 12 (position 58,327,459–58,336,915) (A-allele associated with disease, OR (CI) 1.25 (1.04–1.50); *P*_G_ = 0.02) using ANOVA (*P*_I_ = 0.02; Table 3). The *P* value equaled 0.04 with Bonferroni correction considering that we performed two ANOVAs. HERV-K106 contains a 293-bp ‘deletion’ in *env* which is the signature of all type I HERV members. The HERV-K119 locus exists as a full-length HERV-K. The distribution of risk of T1DM on the nine possible genotypes of the SNPs also suggests a synergistic effect (results not shown). Supplemental Table 2 and 3 show association of rs7650483/K119 and rs11172544 with T1DM when patients were stratified for the presence of nephropathy. The associations were not influenced by the patient’s nephropathy status.

**Table 3:** Two-way ANOVA of synergy between markers of HERV-K119 and HERV-K106 in T1DM

Tests of between-subjects effects Dependent variable: tstate					
Source	Type III sum of squares	*df*	Mean square	*F*	Sig.
Corrected model	8.143^a^	8	1.018	4.255	0.000
Intercept	89.840	1	89.840	375.591	0.000
rs7650483	1.498	2	0.749	3.131	0.044
rs11172544	0.382	2	0.191	0.799	0.450
rs7650483 × rs11172544	2.792	4	0.698	2.918	***0.020***
Error	426.966	1785	0.239		
Total	1052.000	1794			
Corrected total	435.108	1793			

*P* value for two-way interaction between rs7650483 and rs11172544 is shown in bold and italics

*R* ^2^ = 0.019 (adjusted *R* ^2^ = 0.014)

### Rheumatoid arthritis (RA)

Statistical analyses on the RA cohorts show two SNPs as being associated with RA after Bonferroni correction: rs5993426 near HERV-K encoding in frame *gag* and *pol* (*broken by stop codon TGA/pro*) viral genes on Chr. 22 (location 18,938,674–18,947,848) (G-allele associated with disease, OR (CI) 2.87 (2.07–3.99); *P*_G_ = 7 × 10^−13^; *P*_B_ = 1 × 10^−10^) and rs2096537 near HERV-H [26] encoding *pol* also on chromosome 22 (position 16,611,308–16,615,149) (*P*_G_ = 0.0002; *P*_B_ = 0.02) [C-allele associated with disease, OR (CI) 1.44 (1.13–1.84)]. Analysis of the interaction between the two SNPs (*P*_I_ = 0.003) is presented in Table 4. This interaction could be in principle due to the fact that the loci are located on the same chromosome, but the distance between SNPs is rather long for linkage disequilibrium (>2 Mb) and there was no evidence for linkage disequilibrium in the controls (results not shown). Supplemental Table 4 and 5 show the association of rs5993426/HERV-K and rs2096537/HERV-H with RA when patients were stratified for the presence of anti-CPP (IgG antibodies). The associations were not influenced by the patients’ anti-CPP status. Similar results were obtained when patients were stratified for the presence of rheumatoid factor (results not shown). A possible source of *env* viral encoding locus could be HERV-Fc1, as rs391745 was significantly associated with RA (*P*_G_ = 0.01) although not Bonferroni-resistant.

**Table 4:** Two-way ANOVA of synergy between markers of HERV-K and HERV-H in RA

Tests of between-subjects effects Dependent variable: tstate					
Source	Type III sum of squares	*df*	Mean square	*F*	Sig.
Corrected model	39.431^a^	11	3.585	16.590	0.000
Intercept	8.846	1	8.846	40.938	0.000
rs5993426	0.917	2	0.458	2.121	0.120
rs2096537	3.268	3	1.089	5.042	0.002
rs5993426 × rs2096537	3.954	5	0.791	3.660	***0.003***
Error	267.933	1240	0.216		
Total	710.000	1252			
Corrected total	307.364	1251			

*P* value for two-way interaction between rs5993426 and rs2096537 is shown in bold and italics

*R* ^2^ = 0.128 (adjusted *R* ^2^ = 0.121)

## Discussion

Significant advances have been made in dissecting the etiology of autoimmunity over the past decade, both in identifying genetic risk factors, understanding the cellular basis for their effect and describing interplay of environmental components. Particularly, viral infections have been proposed to trigger these diseases on a background of genetic predisposition. Strong associations between the HLA region and autoimmune disease have been established over the past half century [27].

Moving association studies from single-locus analysis to wider gene interactions analysis seems crucial in the continued search for etiologies in complex human diseases. In present investigation, we focused on endogenous retroviral loci and their genetic interactions. Our aim in the present work was the analysis of polymorphisms in or nearby viral loci with at most two breaks in the reading frame of one or more viral protein, and their association with susceptibility to AID. The list of loci was originally based on NCBI 37.1 but was later supplemented with the loci HERV-K113 and HERV-K115 that are only present in certain humans. In each of the three tested diseases (MS, RA, and T1DM), at least one SNP withstood Bonferroni correction (rs391745, rs5993426, and rs7650483, respectively), suggesting that endogenous retrovirus may play a role in all three. Additional retroviral loci seemed to contribute, too (rs2435031, rs2096537, and rs11172544). Although we have observed some overlap, most loci occurred only in one particular disease. Thus, different endogenous retroviruses would conceivably determine the resulting disease phenotype.

Our further analysis took advantage of the fact that association terms and product terms generally are statistically independent; i.e., even if we optimize for association, we can only expect high interaction if there is a biological synergy. In MS, the three-way interaction of SNPs near HERV-Fc1 rs391745, HERV-K13 rs2435031, and HLA rs2135388 in particular suggests that the viral regions are relevant to this disease. Importantly, the statistical synergy indicates that the three loci *together* may contribute to MS. We are not simply observing additive effects, in which each locus influences MS separately. The actual mechanism by which HERV-Fc1 and HERV-K13 might influence the immune regulation of HLA and eventually cause MS development still remains to be elucidated. One possibility is that proviral elements, e.g., antigens mediating molecular mimicry effects, on a background of deregulated immune factors could trigger an autoimmune response. Recombination between retroviruses depends on the co-packing of different viral RNAs into the same particle [28]. Thus, some form of complementation is a prerequisite for recombination. HERV-Fc1 has the potential to express a full-length Env product of 584 aa, and a Gag product of 470aa. In silico analysis of predicted ORFs in the HERV-Fc1 sequence shows that the first two stop codons in the region of *gag*–*pol* boundary are separated by only 165 bp. Thus, if the first stop codon is indeed a premature termination signal in *gag*, this would cause deletion of the last 55 aa in the C-terminal of a protein with an original size of 526aa (unpublished data). It is uncertain whether this Gag corresponds to full-length or a premature termination product which lacks 55aa. The HERV-Fc1 *pol* frame is disrupted by several mutations, whereas HERV-K13 was included in this study based on its near-open *pol* gene, which is disrupted by only one frame shift. Interestingly, HERV-K13 also has an ORF for a 901 aa long Gag protein.

Similar genetic considerations apply to the observed associations and two-way interactions observed in the other diseases. In RA, we observed interaction of two SNPs near HERV-K encoding *gag* and *pol* (*stop codon TGA/pro*) and HERV-H encoding *pol*, rs5993426 and rs2096537, respectively. In T1DM, we have shown a synergy between two SNPs in a close proximity to human-specific full-length HERV-Ks loci, HERV-K106 and the HERV-K119. HERV-K106 has been identified as having the highest probability of being the youngest full-length endogenous retroviruses in the human genome with no sequence difference between its LTRs [29]. Moreover, the presence of the 293-bp *env* deletion in HERV-K106 which is characteristic in multiple HERV-K type I members suggests that this deletion may have been present in the infectious ancestral precursors of these viruses. A highly polymorphic HERV-K119 locus possesses intact gene components, which indicates that it has a potential to encode the functional proteins. Interestingly, Shin and others [29] suggested that the HERV-K119 pattern of polymorphisms is different from that of the other elements of HERV-K family (e.g., HERV-K113 or HERV-K115) and that the HERV-K119 locus exists as either a full-length HERV-K or a solitary LTR. In this case, a possible role for the HERV in autoimmunity with a special focus on T1DM is inadvertently linked to the presence of the ORFs in susceptible individuals. If so, then one would expect that the SNP is linked to the full-length or the truncated allele. Otherwise, the SNP might be linked to other genes or to the promoter activity of the HERV-K119 LTR, which scenario is the case remains to be determined.

ERVs can recombine to generate viruses with new infectious properties, as well established in mice, but also seen in other species. Our genetic data are currently exclusively based on genetic association and cannot as such predict any functional relationship beyond the presence or absence of SNPs. Nevertheless, statistical associations are most easily explained by a functional relation, and it is therefore pertinent to pursue the potential molecular interactions between viruses. As such, it is possible that HERV-K loci coding for full-length HERV-K genomes have all the features necessary for replication as a viral particle. Moreover, it is also plausible that complementation and/or recombination among the multiple full-length HERV-Ks proviruses or even members of others HERVs families in the human genome could lead to emergence of replication-capable viruses. Among 29 human-specific HERV-K insertion events, 17 are full-length human-specific insertions with all sequences required for HERV-K replication. Excluding gene conversion in the host genome, we speculate that recombination/complementation might occur during reverse transcription of two co-packaged RNA genomes. In fact as described, the rarity of complementation *in trans* among HERV families is surprising given that retroviral replication involves the obligate co-packaging of two viral mRNAs within the same viral particle [30]. We and others speculate that this is caused by a low probability that more than one element was expressed in the same cell at the same time. Several HERVs have been implicated in autoimmune disorders based on the presence of activated HERVs molecules.

An intriguing question remains about how endogenous retroviral recombinants could induce autoimmunity? Presumably in a similar manner to exogenous animal and human viruses, the mechanism is not understood. First of all, activity of viral regulatory regions could potentially affect expression of nearby elements such as immune regulatory genes, leading to aberrant regulation. Alternatively, expression of HERV genes themselves has the potential to activate the immune system. The innate immune system serves as the first defense mechanism during infection of exogenous viruses, via detection of pathogen-associated molecular patterns (PAMPs) through pattern recognition receptors (PRRs), activating expression cascade of proinflammatory cytokines. The envelope SU-domain of HERV-W has been shown to activate the PRR CD14 and Toll-like receptor (TLR) 4 with stimulation of interleukin (IL)-1β, IL-6, and TNFα [31], and HERV-K dUTPase can activate a number of ILs and interferon (IFN)-γ through TLR2 [32]. The proinflammatory response induced by the innate immune system also contributes to prime the adaptive immune system. The adaptive immune system may be activated by viral proteins acting as superantigens (SAgs), leading to massive non-specific T cell activation and cytokine release [33, 34]. We speculate that virus particles stimulate the innate immune system through the receptor proteins *TRIMs*, *STING*, and *BST*-*2.* The discovery of TRIM5 triggering of innate immune signaling upon binding the capsid of a nucleic acid-devoid retrovirus-like particles (RVLPs) and a similar induction of innate immune signaling following virion restriction by the host factor tetherin, could be particularly interesting in relation to our present study and our previous observations of genetic associations between TRIM5 and BST-2 markers and the occurrence of MS [35]. This could actually provide mechanisms by which activated HERVs are a source of such RVLPs. Perhaps, formation of particles is enough, and reproductive infection by the particles is not even necessary, as long as the expression is high enough. Later, the reaction might spill over into the adaptive immune system and cause it to respond to cellular components.

One possible *caveat* in our study is the fact that we have used SNPs as proxies for the viral loci and it is among the SNPs we have found statistical associations and interactions. Although the SNPs are very close to the loci, it remains a possibility that we are observing the effect of other genes close by. Specifically, there is a HERV-H-related sequence near HERV-K13, which could be relevant to MS. Additionally, none of the viral loci was recognized as disease relevant in high-density genome-wide association studies. SNPs located near or inside retroviral elements are poorly represented in the predesigned Illumina chips generally used for large-scale GWAS. These chips were deliberately designed to focus on single-copy sequences. Also, although in sheer numbers the GWAS have more strength than our data sets, the large number of comparisons made by GWAS necessitates large compensations for the multiplicity of testing. Therefore, we believe that an approach dedicated to investigating endogenous retroviruses such as ours has definite advantages.

## Electronic supplementary material

Supplementary material 1 (DOCX 19 kb)

Supplementary material 2 (DOCX 14 kb)

Supplementary material 3 (DOCX 14 kb)

Supplementary material 4 (DOCX 14 kb)

Supplementary material 5 (DOCX 14 kb)
