# Supplementary material for: Are human endogenous retroviruses triggers of autoimmune diseases? Unveiling associations of three diseases and viral loci
Source: Immunol Res. 2015 Jun 20;64:55–63. doi: 10.1007/s12026-015-8671-z (PMC4726719; doi:10.1007/s12026-015-8671-z)
Supplement: Supplementary file 1 — Supplementary material 1 (DOCX 19 kb) [file 12026_2015_8671_MOESM1_ESM.docx]

**Supplemental Table 1 A list of 51 HERV loci able, with one or two mutations per viral genome, to encode one or more of viral proteins.**

| **Chromosome number** | **HERV family** | **Chromosomal location (NCBI Annotation 106)** | **Coding or near-coding genes** |
| --- | --- | --- | --- |
| 1 | HERV-K | 75,377,086 - 75,383,468 | *gag* |
| 1 | HERV-K | 155,626,666 - 155,635,845 | *pol* |
| 1 | HERV-K | 160,690,785 - 160,700,016 | *pol env* |
| 1 | HERV-H | 183,702,773 - 183,709,310 | *gag* |
| 2 | HERV-9 | 33,053,541 - 33,056,328 | *gag* |
| 2 | HERV-K | 129,961,965 - 129,965,044 | *env* |
| 2 | HERV-H | 154,867,229 - 154,874,560 | *env* |
| 2 | HERV-H | 165,707,552 - 165,716,198 | *gag pol env* |
| 3 | HERV-K | 9,847,662 - 9,854,552 | *Gag* |
| 3 | HERV-K | 101,691,893 - 101,701,015 | *Gag pol* |
| 3 | HERV-K | 113,024,277 - 113,033,435 | *Gag env* |
| 3 | HERV-K | 130,443,990 - 130,453,404 | *Gag pol* |
| 3 | HERV-FH | 151,806,149 - 151,812,455 | *Gag* |
| 3 | HERV-H | 166,822,581 - 166,829,713 | *Env* |
| 4 | HERV-K | 3,977,324 - 3,986,912 | *Pol* |
| 4 | HERV-17 | 8,422,401 - 8,427,765 | *Pol* |
| 5 | HERV-K | 30,486,653 - 30,496,098 | *Gag pol env* |
| 5 | HERV-K | 34,460,958 - 34,468,537 | *Env* |
| 5 | HERV-K | 156,657,706 - 156,666,885 | *Pol* |
| 6 | MER50 | 11,102,754 - 11,112,217 | *Env* |
| 6 | HERV-H | 40,861,398 - 40,868,224 | *Env* |
| 6 | HERV-H | 77,658,021 - 77,666,441 | *Env* |
| 6 | HERV-K | 77,716,945 - 77,726,366 | *Env* |
| 7 | HERV-K | 4,582,426 - 4,600,400 | *Env* |
| 7 | HERV3 | 64,990,324 - 64,999,936 | *Env* |
| 7 | HERV17 | 92,467,999 - 92,475,579 | *Env* |
| 8* | HERV-K115 |  | *Gag pol env* |
| 9 | HERV-H | 121,790,002 - 121,796,769 | *env* |
| 10 | HERV-H | 25,772,543 - 25,774,917 | *Gag* |
| 10 | HERV-K | 99,820,812 - 99,827,988 | *Pol* |
| 11 | HERV-H | 94,345,725 - 94,351,415 | *Gag* |
| 11 | HERV-K | 101,695,063 - 101,704,528 | *Env* |
| 11 | HERV-H | 118,717,034 - 118,731,855 | *Env* |
| 12 | HERV-H | 18,195,661 - 18,203,727 | *Env* |
| 12 | HERV-K | 58,327,459 - 58,336,915 | *Gag env* |
| 16 | HERV-K | 2,660,262 - 2,670,537 | *Pol* |
| 19 | HERV-S | 20,361,622 - 20,371,352 | *Env* |
| 19 | HERV-K | 21,880,252 - 21,890,542 | *Gag pol* |
| 19* | HERV-K113 |  | *Gag pol env* |
| 19 | HERV-K | 27,637,590 - 27,646,453 | *Gag pol env* |
| 19 | MER50 | 53,007,847 - 53,013,824 | *Gag* |
| 22 | HERV-H | 16,611,308 – 16,615,149 | *Gag* |
| 22 | HERV-K | 18,938,674 – 18,947,848 | *Gag pol* |
| 22 | HERV-K | 19,934,438 - 19,940,687 | *gag* |
| X | HERV-H | 65,361,589 - 65,365,966 | *Pol* |
| X | HERV-HF | 72,220,391 - 72,229,908 | *Env* |
| X | HERV-E | 93,429,668 - 93,438,491 | *Gag env* |
| X | HERV-Fc1 | 97,841,482 - 97,849,424 | *Gag env* |
| X | HERV-K | 105,666,189 - 105,669,044 | *Env* |
| X | HERV17 | 107,052,131 - 107,054,871 | *Env* |
| X | HERV-K | 149,719,916 - 149,727,680 | *Env* |

*only present in certain humans
