# Supplementary material for: Are human endogenous retroviruses triggers of autoimmune diseases? Unveiling associations of three diseases and viral loci
Source: Immunol Res. 2015 Jun 20;64:55–63. doi: 10.1007/s12026-015-8671-z (PMC4726719; doi:10.1007/s12026-015-8671-z)
Supplement: Supplementary file 2 — Supplementary material 2 (DOCX 14 kb) [file 12026_2015_8671_MOESM2_ESM.docx]

**Supplemental Table 2 Association of rs7650483/K119 on chromosome 3 with T1DM patients when stratified for Nephropathy**

| Group | Persons | CC | CT | TT |
| --- | --- | --- | --- | --- |
| 1 | CONTROLS | 103 | 284 | 382 |
| 2 | CASES - Nephropathy | 40 | 160 | 210 |
| 3 | CASES + Nephropathy | 27 | 156 | 201 |
| 4 | All CASES | 86 | 432 | 585 |
| Comparison of Groups | OR (95%CI)  C-allele vs T-allele | P_Allele_ |  |  |
| 2 vs 3 | 1.10 (0.88 – 1.37) | 0.40 |  |  |
| 2 vs 1 | 0.89 (0.74 – 1.06) | 0.19 |  |  |
| 3 vs 1 | 0.80 (0.66 – 0.97) | 0.026 |  |  |
| 4 vs 1 | 0.81 (0.70 – 0.93) | 0.003 |  |  |
