# Supplementary material for: Are human endogenous retroviruses triggers of autoimmune diseases? Unveiling associations of three diseases and viral loci
Source: Immunol Res. 2015 Jun 20;64:55–63. doi: 10.1007/s12026-015-8671-z (PMC4726719; doi:10.1007/s12026-015-8671-z)
Supplement: Supplementary file 3 — Supplementary material 3 (DOCX 14 kb) [file 12026_2015_8671_MOESM3_ESM.docx]

**Supplemental Table 3 Association of rs11172544/K106 on chromosome 12 with T1DM patients when stratified for Nephropathy**

| Group | Persons | AA | AG | GG |
| --- | --- | --- | --- | --- |
| 1 | CONTROLS | 17 | 266 | 508 |
| 2 | CASES - Nephropathy | 19 | 126 | 235 |
| 3 | CASES + Nephropathy | 20 | 117 | 229 |
| 4 | All CASES | 58 | 347 | 611 |
| Comparison of Groups | OR (95%CI)  A-allele vs G-allele | P_Allele_ |  |  |
| 2 vs 3 | 1.01 (0.79 – 1.29) | 0.95 |  |  |
| 2 vs 1 | 1.18 (0.95 – 1.46) | 0.14 |  |  |
| 3 vs 1 | 1.17 (0.94 – 1.45) | 0.16 |  |  |
| 4 vs 1 | 1.26 (1.07 – 1.48) | 0.005 |  |  |
