# Supplementary material for: Are human endogenous retroviruses triggers of autoimmune diseases? Unveiling associations of three diseases and viral loci
Source: Immunol Res. 2015 Jun 20;64:55–63. doi: 10.1007/s12026-015-8671-z (PMC4726719; doi:10.1007/s12026-015-8671-z)
Supplement: Supplementary file 4 — Supplementary material 4 (DOCX 14 kb) [file 12026_2015_8671_MOESM4_ESM.docx]

**Supplemental Table 4 Association of rs2096537/H with RA when patients were stratified for anti-CCP.**

| Group | Persons | AA | AC | CC |
| --- | --- | --- | --- | --- |
| 1 | CONTROLS | 14 | 129 | 391 |
| 2 | CASES – Anti-CCP | 0 | 47 | 158 |
| 3 | CASES + Anti-CCP | 1 | 74 | 283 |
| 4 | CASES Total | 1 | 141 | 527 |
| Comparison of Groups | OR (95%CI)  A-allele vs C-allele | P_Allele_ |  |  |
| 2 vs 3 | 1.09 (0.74 – 1.60) | 0.7 |  |  |
| 2 vs 1 | 0.75 (0.53 – 1.06) | 0.11 |  |  |
| 3 vs 1 | 0.69 (0.51 – 0.92) | 0.01 |  |  |
| 4 vs 1 | 0.69 (0.54 – 0.88) | 0.003 |  |  |
