# Supplementary material for: Are human endogenous retroviruses triggers of autoimmune diseases? Unveiling associations of three diseases and viral loci
Source: Immunol Res. 2015 Jun 20;64:55–63. doi: 10.1007/s12026-015-8671-z (PMC4726719; doi:10.1007/s12026-015-8671-z)
Supplement: Supplementary file 5 — Supplementary material 5 (DOCX 14 kb) [file 12026_2015_8671_MOESM5_ESM.docx]

**Supplemental Table 5 Association of rs5993436/K with RA when patients were stratified for anti-CCP.**

| Group | Persons | GG | GT | TT |
| --- | --- | --- | --- | --- |
| 1 | CONTROLS | 0 | 48 | 352 |
| 2 | CASES – Anti-CCP | 0 | 56 | 138 |
| 3 | CASES + Anti-CCP | 0 | 101 | 229 |
| 4 | CASES Total | 0 | 194 | 432 |
| Comparison of Groups | OR (95%CI)  G-allele vs T-allele | P_Allele_ |  |  |
| 2 vs 3 | 0.93 (0.66 – 1.33) | 0.7 |  |  |
| 2 vs 1 | 2.64 (1.76 – 3.96) | 4*10^-7^ |  |  |
| 3 vs 1 | 2.82 (1.97 – 4.05) | 5*10^-10^ |  |  |
| 4 vs 1 | 2.86 (2.06 – 3.98) | 3*10^-12^ |  |  |
